# Applications of miRNAs in cardiac development, disease progression and regeneration

**DOI:** 10.1186/s13287-019-1451-2

**Published:** 2019-11-21

**Authors:** Jeremy Kah Sheng Pang, Qian Hua Phua, Boon-Seng Soh

**Affiliations:** 1 Disease Modeling and Therapeutics Laboratory, A*STAR Institute of Molecular and Cell Biology, 61 Biopolis Drive Proteos, Singapore, 138673 Singapore; 2 Department of Biological Sciences, National University of Singapore, Singapore, 117543 Singapore; 3 Key Laboratory for Major Obstetric Diseases of Guangdong Province, The Third Affiliated Hospital of Guangzhou Medical University, Guangzhou, 510150 China

**Keywords:** MicroRNA, Stem cell niche, Cardiac development, Cardiovascular progenitors, Cardiac regeneration and development

## Abstract

Development of the complex human heart is tightly regulated at multiple levels, maintaining multipotency and proliferative state in the embryonic cardiovascular progenitors and thereafter suppressing progenitor characteristics to allow for terminal differentiation and maturation. Small regulatory microRNAs (miRNAs) are at the level of post-transcriptional gene suppressors, which enhance the degradation or decay of their target protein-coding mRNAs. These miRNAs are known to play roles in a large number of biological events, cardiovascular development being no exception. A number of critical cardiac-specific miRNAs have been identified, of which structural developmental defects have been linked to dysregulation of miRNAs in the proliferating cardiac stem cells. These miRNAs present in the stem cell niche are lost when the cardiac progenitors terminally differentiate, resulting in the postnatal mitotic arrest of the heart. Therapeutic applications of these miRNAs extend to the realm of heart failure, whereby the death of heart cells in the ageing heart cannot be replaced due to the arrest of cell division. By utilizing miRNA therapy to control cell cycling, the regenerative potential of matured myocardium can be restored. This review will address the various cardiac progenitor-related miRNAs that control the development and proliferative potential of the heart.

## Introduction

At the cellular level, response to environmental stimuli requires tight regulation of gene expression. This control is spread amongst a number of key players that play regulatory roles at different stages of gene expression. MicroRNAs (miRNAs) are a class of non-coding RNAs (~ 23 nucleotides) that regulate gene expression post-transcription of their associated messenger RNAs (mRNA), termed RNA interference (RNAi). Significant progress in our understanding of miRNA regulation and mechanism has been made within the past decade. To date, miRNAs have been shown to play roles in a large number of biological events, such as embryonic development wherein miRNAs regulate transcription factor expression, setting up morphogen concentration gradients as well as contributing to the dynamic processes of stem cell maintenance, early cell migration and proliferation [1, 2]. Scientific interest in miRNA lies in its contribution to development [3], dysregulation in diseases [4] and therefore potential as a disease biomarker and therapeutic [5, 6]. There are also efforts in pushing RNAi-based pharmaceuticals as a post-transcriptional targeted gene silencing therapeutic [7]. Due to the fine-tuning properties of miRNA, there is a demand to decipher the roles and targets of miRNA in the various biological processes.

**Fig. 1:**
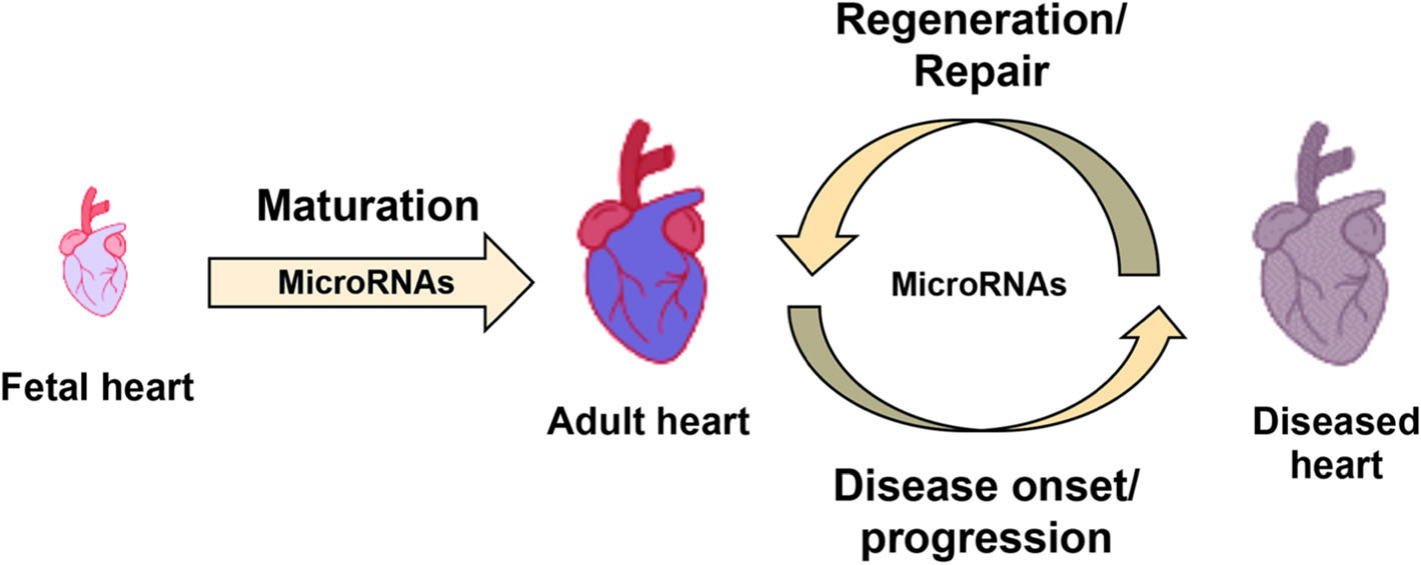
Involvement of microRNAs in the various phases of the heart covered in this review—maturation, disease progression and regeneration

In the field of cardiovascular research, various miRNAs have been shown to contribute to cardiac fate specification and maintenance of cardiac progenitor during development. As would be expected, dysregulated miRNA expression in embryonic cardiovascular progenitors during development would lead to numerous structural congenital heart defects as a result of poorer cell proliferation, migration and specification into appropriate cell types [8]. These structural defects do not repair themselves as the cardiac stem cells terminally differentiate and exit the cell cycle postnatally and no longer proliferate. Thereafter, cardiomyocytes express cardiac-specific miRNAs which maintain the postnatal mitotic arrest [9, 10]. As a result, humans inevitably suffer from increasing risks for heart failure throughout our ever-increasing life expectancy as we accrue heart injuries which do not get repaired. Therefore, further research to better understand the miRNA transcriptome and regulatory pathways in cardiac progenitors are crucial to prevent the development of congenital heart defects. In addition, due to the increasing appeal of regenerative therapeutics for the adult heart, the search for embryonic cardiovascular progenitor miRNAs and adult cardiac-specific miRNAs is a potential breakthrough to generate proliferative progenitor niches via miRNA therapy. A broad schematic overview detailing the various involvements miRNA have in cardiovascular research is shown in Fig. 1. This review briefly summarizes the properties of miRNAs and their mode of action while the significant focus is given to the potential translational applications of miRNAs related to congenital heart disease and adult cardiac tissue regeneration.

## MicroRNAs—biogenesis and mechanism of action

**Fig. 2:**
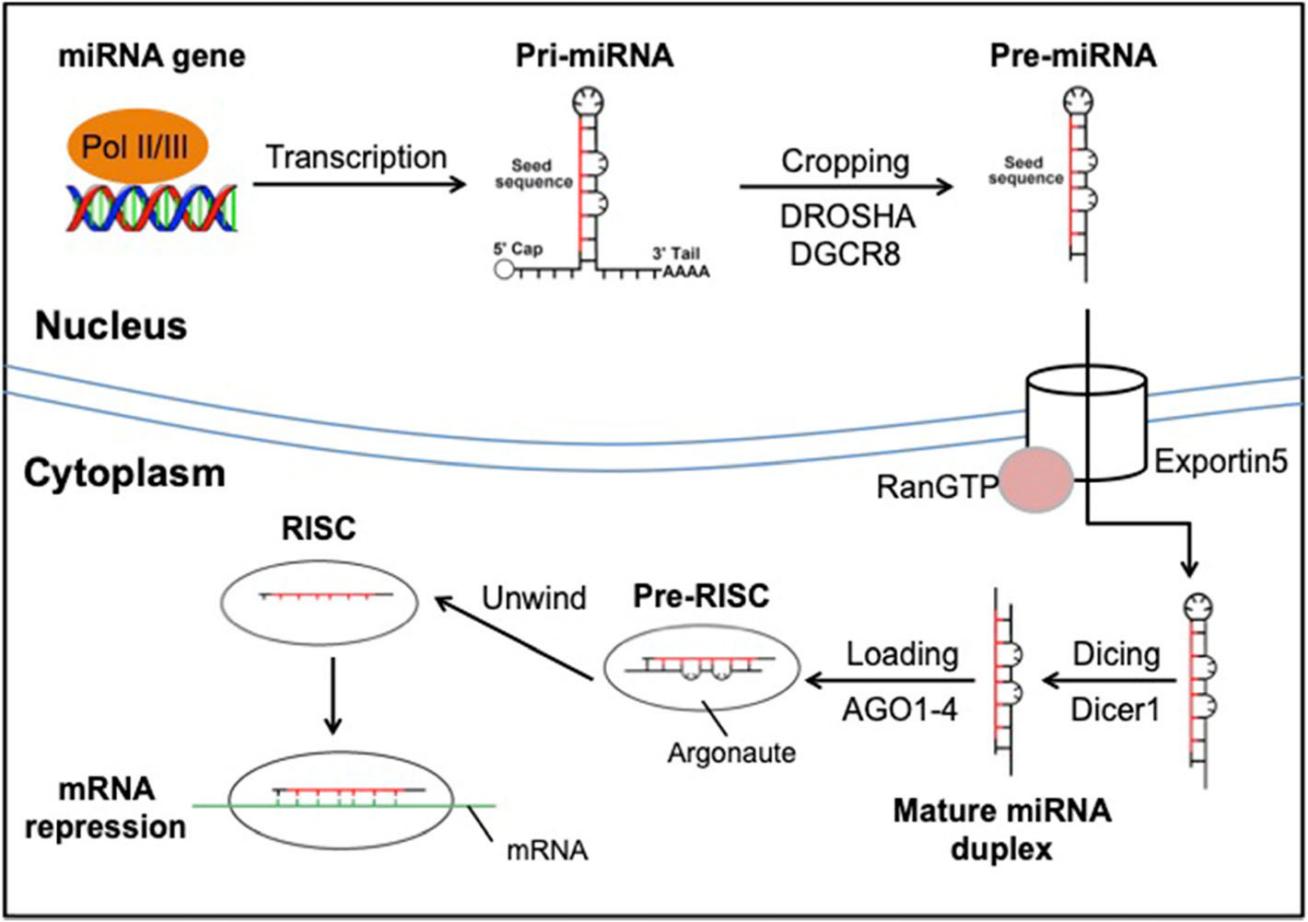
Schematic illustration of miRNA biogenesis

The topic of miRNA biogenesis and mechanism of action has been reviewed extensively as the understanding of the field progressed over the years [11–14]. Within the family of miRNAs, there are variations in biogenesis, but they all eventually lead to a functional ribonucleoprotein complex termed RNA-induced silencing complex (RISC) [14]. Briefly, majority of miRNAs originate from the transcription of their primary miRNA (pri-miRNA) by RNA polymerase II or III followed by cleavage by RNase III Drosha and DGCR8 to their precursors (pre-miRNA) [15]. The pre-miRNAs are thereafter shuttled into the cytoplasm via Exportin5/RanGTP complex to be further processed by RNase III Dicer to produce a mature miRNA duplex [16]. The mature miRNA duplex is then loaded onto an Argonaute family protein and then unwound into the single miRNA guide strand used by the RISC for targeted mRNA silencing. A simplified schematic of miRNA biogenesis is portrayed in Fig. 2.

RISC carries out post-transcriptional silencing via the guide miRNA base pairing to miRNA response element (MRE) sequence on the mRNA [17, 18]. MREs contain complementary base pairs to the seed sequence of the guide miRNA (residues 2–8 at the 5′ end), and while typically found on the 3′-untranslated region (3′ UTR), MREs have also been identified in the 5′ UTR, coding sequences and promoter regions [19, 20]. The RISC:MRE interaction results in either mRNA cleavage via Argonaute (AGO) endonuclease or mRNA decay due to deadenylation and decapping via recruited effector proteins [21, 22]. RISC recruits GW182 family proteins which provide the scaffolding for CCR4-NOT deadenylase and DCP1:DCP2 decapping complexes, rendering the mRNA unstable and eventually degraded 5′-3′ by exoribonuclease 1 [23, 24]. Experimental results have shown that 3′ UTR lengthening increases the number of miRNA binding sites and augments post-transcriptional control of gene expression [25]. On the other hand, shortening of 3′ UTR has been shown to be widespread in cancer cells, resulting in the loss of miRNA-mediated repression [26]. As such, miRNA is able to finely regulate gene expression by reducing levels of available mRNA for translation.

## miRNAs in development

### Embryonic development—pluripotency maintenance and cellular differentiation

Looking at the bigger picture, miRNA control over gene expression has been shown to be crucial for pluripotency maintenance and embryonic development. A detailed review was previously provided by Carnell et al. [27]. Briefly, early research revealed that homozygous *Dicer1* knockout in mice resulted in embryonic lethality and heterozygous mutants stained for Oct4 showed much reduced protein expression, indicating a loss of the stem cell population in the embryo. Later research unveiled key miRNAs highly expressed in embryonic stem cells involved in pluripotency maintenance, such as miRNA-34a and the miR-290 family (miR-291-3p, miR-294, miR-295) [28–30]. Amongst several other miRNA families, the miR-302 and miR-372 clusters that share a similar seed sequence with the miR-290 family have also been used to improve the efficiency of classical induced pluripotent stem cell reprogramming [2, 31].

Further down the pipeline, miRNAs also play a key role in regulating the downstream differentiation and large-scale cell migrations into the different embryonic tissue and organs. Early stages of embryogenesis involve the generation of distinct cell types from a single totipotent cell, via the setting up of morphogen gradients to convey positional information within the embryo [32]. The presence of miR-430, a repressor of both the agonist and antagonist of the Nodal morphogen gradient, in the zebrafish embryo acts as a buffer against transient fluctuations of Nodal, preventing patterning mistakes in the embryo [33]. At the same time, miRNAs can facilitate the decay of existing morphogen mRNAs when they are no longer required. miR-430 also actively degrades *sdf1a* transcripts in all cells such that only cells actively transcribing new *sdf1a* will express it [34, 35]. Therefore, in the context of development, miRNA is crucial for the fine-tuning of morphogen gradients for accurate fate specification and downstream differentiation.

### Heart formation—migration and differentiation of cardiac mesodermal precursors

Coming to the cell population of interest, embryonic heart development similarly requires tight regulation. In the mammal, the heart is the first functional organ to develop with multiple stages in the cardiac developmental timeline. Firstly, mesodermal precursors are specified into cardiovascular progenitors which migrate during gastrulation to form the two heart fields. Subsequently, the cardiac crescent and tubular heart are formed. Lastly, respective cells are recruited to the poles of the heart, and differentiation into the cardiac chambers occur (Abu-Issa 2007). Development of the heart has been extensively studied, uncovering the roles of *Mesp-1/2*, *Hand1/2*, *Wnt/β-catenin* signalling, *Bmp2* and FGFs in inducing cardiac progenitor migration and setting up the heart fields [36–39]. *Nkx2.5* and *Isl1* expressing cardiac progenitor cells proliferate and contribute to the initial formation of the heart tube and eventual chamber structures, conduction channels and nodes [40–43]. It is now known that miRNA machinery is co-opted to repress inhibitors of cardiac differentiation, to enhance expression of mesodermal/cardiac specification genes during cardiac development. Initial research utilized targeted *Dicer* knockouts in mice cardiac tissue, resulting in perturbed cardiac development and various cardiac pathological phenotypes such as hypertrophy, fibrosis, biventricular enlargement and ultimately heart failure [44]. Homozygous deletion of *Dicer* in zebrafish led to embryonic lethality due to heart developmental defects amongst several gastrulation associated issues [45]. These findings confirmed the importance of miRNA in cardiac development.

Further research identified a number of cardiac-specific miRNAs, most notably miR-1 and miR-133, which are highly abundant and conserved in orchestrating cardiac development [46]. In the context of development, both miR-1 and miR-133 repress non-muscle gene expression, suppressing endoderm and ectodermal lineages while promoting mesoderm formation during embryonic stem cell differentiation [47]. Ivey et al. reported that miR-1 inhibits Notch ligand Delta-1 directly and the depletion of Delta-1 from ES cells resulted in the differentiation towards cardiac lineage [48]. Mesodermal precursors are enriched in Mesp1, a transcription factor that activates the transcription of miR-322 and miR-503 clusters resulting in the specification into cardiomyocyte progenitors of the mesodermal cells by repression of Celf family proteins [49]. Thereafter, miR-1 and miR-133 take on antagonistic roles, wherein miR-133 serves to maintain the proliferative capacity of cardiac progenitors and maintaining the progenitor pool while miR-1 drives further differentiation of the progenitors into terminally differentiated myocytes [48]. Mechanistically, miR-133 represses serum response factor and cyclin D2 to promote cardiomyocyte proliferation [48, 50]. Hand2 and Irx5 are two targets of miR-1, containing the MRE in their 3′ UTR. A precise dosage of Hand2 was shown to be crucial for normal cardiomyocyte development. The miR-1-2 overexpression or Hand2 targeted knockout resulted in a similar embryonic lethality phenotype in mice, while miR-1-2 deletion led to a fourfold increase in Hand2 protein levels and homozygous lethality due to ventricular septal defects (VSD) [51, 52]. Mice that survived postnatally exhibited phenotypes associated with electrophysiological defects, with cell lines showing a fivefold increase in Irx5 protein levels, a regulator of ventricular repolarization gradient via repression of Kcnd2 potassium channel.

Other prominent cardiac miRNAs have been identified within the cardiac differentiation lineage, highlighted in Table 1. Cardiac progenitors within the second heart field have been shown to express BMP signals that utilize miR-17-92 cluster to promote myocardial differentiation towards the cardiac outflow tract by repression of *Isl1* and *Tbx1* progenitor gene expression, which contain the MREs in their 3′ UTRs [53]. In mice, miR-130a plays a role in regulating cardiac development by targeting Friend-of-GATA 2 (FOG2) [54]. In zebrafish, miR-138 plays a role in ventricular cardiomyocyte maturation, whereby loss of miR-138 perturbs the morphology of the ventricular cardiomyocyte, disrupting the heart function [55]. Therefore, the precise expression of cardiac miRNAs is required for proper morphological and electrophysiological development of the foetal heart.

**Table 1:** miRNA with known targets and their functions in animal models

miRNA	Animal model	Target	Function	References
miR-1	Mice	Hand2	Downregulate ventricular cardiomyocytes expansion, promote cardiac differentiation	[51]
	Mice	Delta-1	Promotes mesoderm formation, cardiac lineage determination	[48]
	*Drosophila*	Delta	Regulates expansion of cardiac and muscle progenitor cells	[56]
miR-17-92	Mice	Isl1, Tbx1	Regulation proliferation of cardiac progenitors	[53]
miR-130a	Mice	Fog-2	Regulates myocardium growth	[54]
miR-133a	Mice	Serum response factor, cyclin D2	Negatively regulate cardiomyocyte proliferation	[50]
miR-138	Zebrafish	Cspg2	Regulates ventricular cardiomyocyte maturation	[55]

### miRNAs involved in human cardiogenesis

**Fig. 3:**
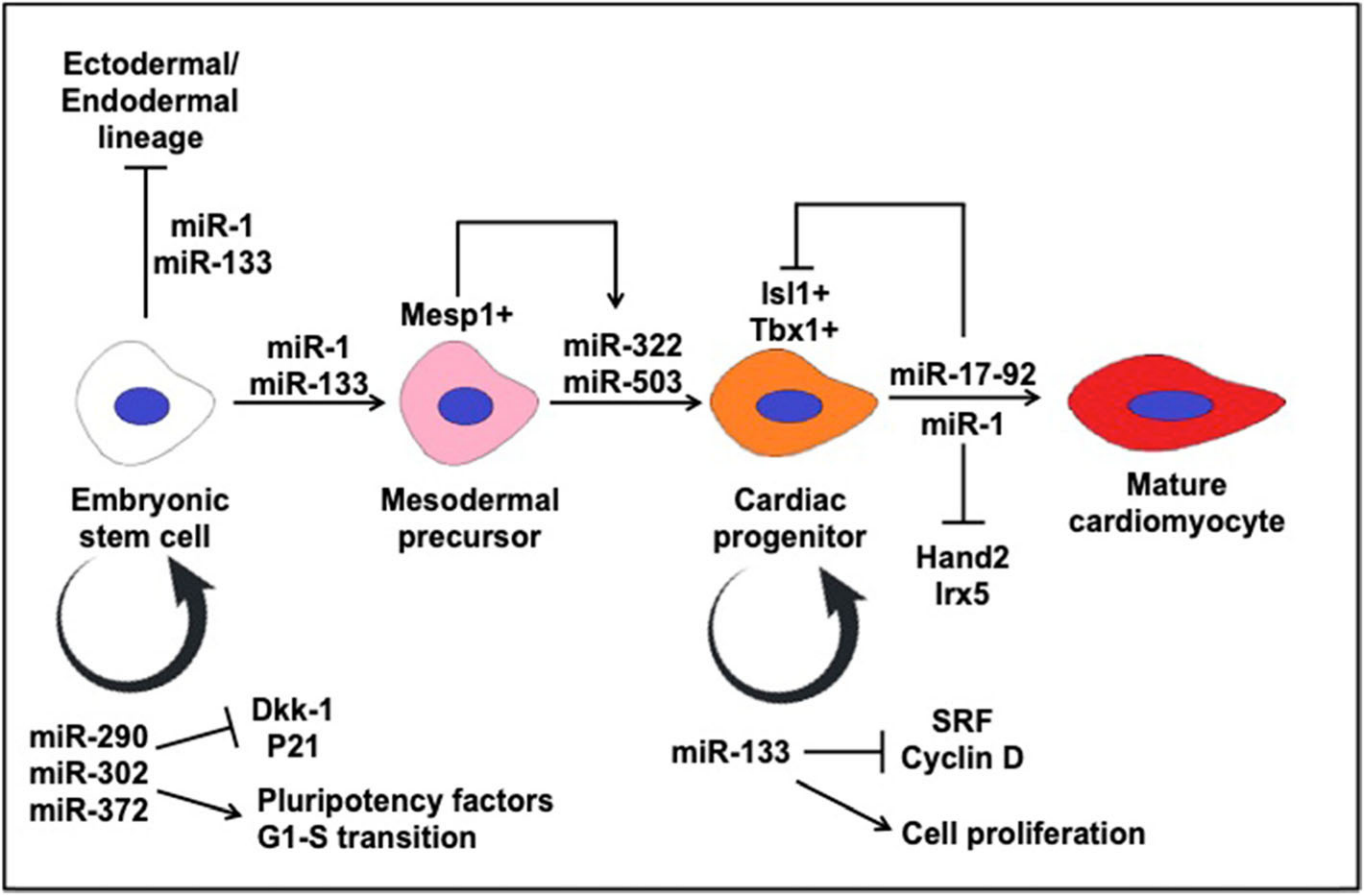
Previously known miRNAs and their associated genes involved in embryonic heart development into cardiomyocytes

A significant amount of our understanding of miRNAs implicated in heart development has been derived from animal models, as portrayed in Fig. 3. However, it is important to note that non-mammalian models such as *Drosophila* or zebrafish do not develop mammalian four-chambered hearts and may not be an accurate depiction of human developmental defects. In reality, there is little characterizing done on miRNAs present in the developing human heart apart from an expression profiling study which has identified upregulated miRNAs likely to be involved in the heart chamber, septum and outflow tract development [57]. There is a need to translate identified miRNAs from animal models to human systems to further our understanding of the gene expression control in human cardiac development. Clinical samples for human heart development are few and far between to come by, and therefore, in vitro models of cardiac development would have to suffice for now. Currently, in vitro human pluripotent stem cell differentiation into the cardiac lineage can be used to model the cardiac development [58].

Using in vitro differentiation of human embryonic stem cells, researchers are able to derive cardiac progenitors, cardiomyocytes, endothelial cells and smooth muscle cells to carry out miRNA profiling. Notably, miR-1 and miR-499 were identified in derived cardiomyocytes, and their role in cardiomyocyte fate commitment was looked into [59, 60]. miR-1 itself has been extensively studied as it is the most specific and abundantly expressed miRNA in the heart [48]. The expression of miR-1 inhibits both the WNT and FGF via repression of FZD7 and FRS2, respectively, promoting cardiomyocyte commitment and repressing endothelial cell fate [59]. Additionally, miR-1 also represses a number of other gene transcripts related to cardiac development or function such as HAND2, HDAC4, GJA1 and KCNJ [61]. Similarly, miR-499 expression enhances the differentiation of cardiac progenitors to cardiomyocytes, possibly through SOX6 inhibition based on the predictions of the seed sequence [61]. Less commonly studied miRNAs such as miR-204, miR-669 and miR-23 have also been shown to promote cardiac progenitor differentiation, but their mechanisms remain to be elucidated [62, 63]. Therefore, future studies looking into the mechanistic targets of cardiac-specific miRNAs in relation to cardiac development and cell fate commitment would be required. These miRNA targets could provide useful information on dysregulated genes during congenital heart defects and could also be potential drug molecules to renew the cardiac progenitor niche in the adult heart for regeneration.

## Dysregulated miRNAs and cardiovascular pathologies

Dysregulation in miRNAs related to cardiac progenitors and development will inevitably lead to structural defects of the heart. In the past, diagnosis of cardiovascular diseases used to be mainly through echocardiography or clinical syndromes. However, molecular biology advancements have uncovered diagnostic biomarkers which could be potentially used to detect cardiac diseases, providing insights into the possible pathological mechanisms underlying these diseases [64]. Identification of miRNAs related to cardiac progenitors and cardiogenesis is the first step to uncovering effects of miRNA dysregulation in congenital heart defects. On the other hand, cardiac-specific miRNAs in the adult hearts can also be dysregulated and cause cardiovascular pathologies such as left ventricular hypertrophy, myocardial infarction and arrhythmias [65–67]. The accumulation of these cardiac diseases results in the structural and functional impairment of the heart, predisposing the risk to heart failure. Translational research utilizing miRNA for therapy relies on identifying and characterizing miRNAs associated with diseases. This section covers the miRNA dysregulation in congenital heart disease and the eventual onset of structural defects in the adult heart leading to myocardial infarction and heart failures.

### MicroRNAs in congenital heart diseases

Congenital heart diseases (CHDs) encompass a broad range of phenotypes resulting from cardiac structural defects at birth and are the most common type of birth defect present amongst newborns, accounting for about 25% of infant morbidity within the first month [68]. As the process of cardiogenesis is an intricate process involving various pathways, origins of the numerous CHDs such as VSD, tetratology of fallot (TOF) and syndromic CHD are typically multifactorial. However, one main cause of CHD stems from an impaired balance between cardiomyocyte proliferation and apoptosis, of which the cardiac development-related miRNAs discussed in the previous section clearly play a part. Table 2 contains studies that have identified dysregulated miRNAs linked with the occurrence of these cardiac deformities.

**Table 2:** miRNAs with known associations to cardiovascular diseases

miRNA	Animal models	Targets	Level of expression	Cardiovascular diseases	References
miR-1	Mice	Hand2	Downregulated	Ventricular septal defect	[51]
	Mice	Mef2a, calmodulin	Downregulated	Cardiac hypertrophy	[73, 74]
	Rat	Kcnj2	Upregulated	Atrial fibrillation	[75, 76]
			Downregulated	Ventricular arrhythmia	[77]
	Human	GJA1, SOX9	Downregulated	Ventricular septal defect	[59]
miR-21	Human	–	Upregulated	Myocardial infarction	[65]
miR-23a	Mice	Foxo3a	Upregulated	Cardiac hypertrophy	[67]
miR-29b	Human	–	Upregulated	Myocardial infarction	[65]
miR-126	Mice	Spred1, PI3KR2a	Downregulated	Defective angiogenesis, leaky and fragile vessels	[78]
miR-181	Human	BMPR2	Upregulated	Ventricular septal defect	[59]
miR-195	Human	CHECK1	Upregulated	Ventricular septal defect	[9]
miR-328	Mice, rat	Cacna1c, Cacnb2	Upregulated	Atrial fibrillation	[79]
miR-421	Human	SOX2	Upregulated	Tetralogy of fallot	[80]

In an effort to study TOF, O’Brien et al. compared the non-coding RNA (which includes miRNAs) expression level between non-syndromic TOF infants and healthy infants. From the analysis, approximately 61 miRNAs involved in cardiac development pathway were reported to exhibit an altered expression in the right ventricular myocardium of TOF infants as compared to healthy infants [8]. TOF is a cardiac pathology that combines four congenital abnormalities—overriding aorta, pulmonary valve stenosis, right ventricular hypertrophy and VSD.

VSD itself has been well studied in relation to miRNAs. To date, several animal studies revealed the involvement of miR-1, miR-133a and other miRNAs in the manifestation of VSD. As mentioned previously, 50% of miR-1-2 knockout mice suffered from embryonic lethality, largely due to VSD and chamber dilation as a result of dysregulated Hand2 levels [51, 52]. Consistent with this, VSD patients were found to exhibit reduced miR-1-1 which was closely associated with the upregulated expression of *GJA1* and *SOX9*. The upregulation of both genes is correlated with VSD incidence, implicating them in VSD pathogenesis [59].

Similarly, miRNA dysregulation was also found to associate with syndromic congenital heart diseases such as Down syndrome. Down syndrome is characterized by trisomy 21, and about 40–60% of babies born with it also suffer from CHD, making it one of the most common genetic syndrome of CHD. Five miRNAs, present on chromosome 21—mir99a, let-7c, miR-125b-2, miR-155 and miR-802—were identified to be highly expressed in cardiac tissue of patients suffering from Down syndrome [69]. Therefore, cardiac development can be negatively impacted by the dysregulation of miRNAs in cardiac tissue, leading to CHD development.

### Distinct miRNA signatures of heart pathologies

Each of the congenital heart diseases described is structural defects of the heart that can develop via impairment of various cardiomyocytes proliferation or migratory pathways. As a result, the different pathologies express distinct miRNA transcriptomes alluding to the affected pathways. The distinct transcriptome landscape was evident from a genetic profiling conducted on 67 patients by Ikeda et al., based on three heart pathologies—ischemic cardiomyopathy, dilated cardiomyopathy and aortic stenosis. Forty-three out of 87 miRNAs were expressed differentially in at least 1 of the disease group [70]. Additionally, Ellis et al. analysed the miRNA levels of a group of patients suffering from heart failure and chronic obstructive pulmonary disease. miR-103, miR-142-3p, miR-30b and miR-342-3p were found to be expressed at a significantly lower level in heart failure as compared to the other groups [71]. Although the knowledge of the cardiac miRNA transcriptome and their underlying mechanisms have yet to be fully understood, comparing miRNA coupled with mRNA expression profiles could be a potential diagnosis tool used to discriminate different cardiac pathologies, thereby helping us to better understand the roles of miRNA in the mechanism and progression of the disease. As such, it is worth exploring disease-specific dysregulated miRNAs to understand disease manifestation and possibly uncovering a drug target to normalize miRNA expressions.

### Cardioprotective and biomarker miRNAs in heart failure

Extending our knowledge of dysregulated miRNAs can plausibly help to fix the structural defects of the heart. However, another pertinent issue with cardiac tissue is its lack of regenerative capacity. Cardiac cell death occurs over the human lifespan and can be accelerated due to cardiovascular complications and myocardial infarction. Acute myocardial infarction is characterized by large-scale cardiac cell death, resulting in a scarred heart with impaired cardiac contractility and remodelling of the left ventricle. Artificially altering miRNA levels can confer a cardioprotective effect as shown by Gu et al. wherein miR-21 overexpression in mice reduced myocardial infarction levels by 36.9% and lowered levels of apoptosis [72]. In addition, miR-21 was also able to enhance the viability and proliferation of murine cardiac stem cells post-myocardial infarction by inhibiting PTEN and activating the PI3K/Akt pathway [72].

The accumulation of cardiac tissue damage and myocardial scarring over time add increasing mechanical burden to the heart. As heart failure is the leading cause of death, there is a demand for biomarkers to aid in heart failure risk assessments. In this regard, certain miRNA expression levels have recently been identified to be differentially expressed in heart failure cases. The miRNA transcriptome of a failing heart was found to be highly similar to foetal cardiac tissue, with more than 80% of both induced and repressed miRNAs regulated similarly [66]. In addition, the authors confirmed that the upregulated miRNAs of the failure heart are sufficient to initiate the shift of expression patterns towards a failure heart. Transfection of healthy adult rat cardiomyocytes with these upregulated miRNAs induced cellular hypertrophy and initiated the shift towards foetal gene expression programmes seen likewise in the failing heart [66]. Therefore, the presence of these miRNAs could foreshadow heart failure, acting as useful biomarkers. Table 2 contains a list of miRNAs with altered levels of expression in different cardiovascular diseases that could be potential biomarkers.

## Therapeutic potential of miRNAs in heart failure

### Versatility of synthetic miRNA control of gene expression

As summarized in the previous sections, miRNAs have been shown to play roles in cell cycling and proliferation as well as the onset of cardiac pathologies and therefore can potentially be a therapeutic option. When considering methods to control and modify gene expression, post-transcriptional silencing via miRNA treatment is highly attractive. Apart from post-transcriptional silencing, gene expression can be altered by two main methods. Firstly, transcription of target genes can be reduced via epigenetic modifications, reduction of transcription factor levels or entire gene knockouts [81–84]. Secondly, post-translational silencing by inhibition or degradation of translated protein will also effectively reduce gene expression levels [83, 85]. While these other methods are very useful for shutting down expression, they are not as versatile as miRNA options. Drugs that inhibit or degrade proteins or transcription factors are typically small molecules, requiring large-scale screenings to identify the molecules with the desired effect [86]. Some protein targets might even be part of the undruggable proteome, with no good binding pockets for specific small molecule interactions [87]. Current drugs that make epigenetic modifications on DNA are also small molecules that require screening for and are typically unspecific in their interactions, typically causing global modifications in the genomic DNA of targeted cell types [88]. Targeted gene mutagenesis is an up and coming therapeutic option, having the first CRISPR-based gene editing product moving into clinical trials in the USA and Europe only recently [89]. Gene editing as a therapeutic is not comparable to transient methods to alter gene expression as it is a permanent option, envisioned to treat congenital diseases resulting from faulty protein products.

MicroRNAs, on the other hand, are more versatile and offer a transient handle on gene expression levels. Once miRNAs linked to a potential drug target have been identified, synthesizing complementary oligonucleotide sequences is trivial and acts as a good start point to develop drugs for treatment. Current miRNA-based drugs utilize stable nucleic acid analogues, termed as locked nucleic acids, to generate an antisense oligonucleotide sequence to target mRNA sequences, acting as synthetic miRNA sequences to exert gene silencing [90]. New miRNA-based therapeutics are relatively straightforward to develop, requiring only the identification of miRNA oligonucleotide sequences of the gene targets. During heart failure, silencing of miR-208a, a cardiac-specific miRNA, has been shown to prevent pathological remodelling and myosin switching, enhancing cardiac function [91]. MED13 is one target of miR-208 that has been identified as a metabolic regulator and insulin sensor in mice, contributing to homeostasis [92]. As such, an anti-miR-208 therapeutic drug (MGN-9103) is being developed to combat heart failure, obesity and diabetes [7]. In this manner, regulatory control of any therapeutic gene target can be established by synthesizing its associated miRNA or anti-miRNA oligonucleotide sequences.

### Induction of cardiac regeneration by miRNAs

One main issue with the adult heart is its innate lack of proliferative and regenerative capacity, resulting in damage to myocardial tissue accumulating as a result of ageing, increasing heart failure risks over time. In the context of this review, it is relevant to look at the potential of miRNA therapy in restoring the proliferative capacity of matured myocardium.

Researchers have utilized animal models to show that miRNAs are able to induce regeneration in other tissue types—miR-124 and miR-302 induced regeneration in the brain and lung tissue, respectively [93, 94]. Similarly, an increasing number of miRNAs have been identified to regulate cardiomyocyte proliferation and cardiac function. In mice models, postnatal upregulation of miR-128 and miR-15 family was identified as contributors to cell cycle arrest in the postnatal heart [9, 10]. Subsequent deletion of miR-128 activated cyclin E- and CDK2-positive cell cycle regulators, while inhibition of miR-15 family similarly increased adult cardiomyocyte proliferation, both improving cardiac function after myocardial infarction [10, 95]. On the other hand, the miR-302-367 family is present in the embryonic mouse heart and lost in the postnatal/adult heart. The miR-302-367 family activates proliferation by repressing the Hippo pathway, and transient application of miR-302-367 mimics led to increased cardiomyocyte proliferation, mass, decreased fibrosis and improved function after injury [96].

In a recent attempt to bring regenerative research closer to human therapy, the Giacca group utilized a more clinically relevant porcine model to show the effects of human miRNA has-miR-199a treatment [97]. Their findings were highly relevant to the field of miRNA therapy as it showcased the potential of miRNA therapy in stimulating cardiac repair, improving contractility and muscle mass while reducing fibrosis. However, additional results revealed that treated pigs later developed lethal arrhythmias and tumour-like clusters of cells. While it is clear that miRNA therapy has enabled the proliferation and repair of the cardiac tissue, there is a need for precise spatial and temporal control of the expression and dosage of the miRNA signal such that aberrant organization and cell growth will not instead cause further damage to the heart. Carrying out additional preclinical research to precisely define the appropriate treatment duration, dosage and locale in models that are increasingly physiologically representative of humans would be a step towards translating it into clinical therapy, but even then, constant monitoring would definitely be necessary to prevent any unexpected cardiac events from occurring.

To make up for the adult human myocardium lacking regenerative capacity, miRNA therapies can potentially enhance regeneration by providing a proliferative niche via the upregulation of cardiac progenitor miRNAs (miR-302-367) or downregulation of cell cycling/proliferation inhibiting cardiac miRNAs (miR128/miR-15). These miRNAs therapeutics could work by inducing dedifferentiation locally wherein the terminally differentiated cardiomyocytes change their gene expression pattern, losing their contractile, electrophysiological and other cardiomyocyte properties, and express genes that enable proliferation and migration characteristic of a more multipotent progenitor. In this case, the adult myocardium undergoing mitotic arrest can be reverted towards the proliferative cardiovascular progenitors within the same lineage to enable tissue repopulation and repair. MGN-1374 is one such drug currently being developed, being an anti-miR targeting the miR-15 family seed region, such as to enable post-myocardial infarction remodelling and induction of cardiomyocyte proliferation [7, 98].

On the other hand, miRNA therapeutics could also utilize the epicardium to encourage myocardium repair. Successful models of regeneration induced cardiomyocyte proliferation and repair in injured myocardium via activation of the epicardial cells [99, 100]. First identified in zebrafish models, the impressive regenerative capacity of the zebrafish heart was linked with a epicardial response to elicit proliferation and wound repair [76]. The response was linked to Wills tumour gene 1 (*Wt1*) expression in the epicardial layer bordering the myocardial infarction, inducing a proliferative and neovascularization response [75, 101]. Wt1+ cells are known to be involved in early cardiac development, present as a sheet of epithelial cells covering the heart with the potential to proliferate and differentiate into the different cardiac lineages [102, 103]. Homozygous epicardial *Dicer* knockout mice result in embryonic lethality due to cardiac defects and impaired coronary vessel development in mice, confirming the relevance of miRNAs in epicardial development [104].

For cardiac repair post-myocardial infarction, Wt1+ epicardial cells consist of multipotent cardiac stem cells that can give rise to de novo cardiomyocytes [105] and vascular progenitor cells contributing to angiogenesis [106], which undergo epithelial-to-mesenchymal transition (EMT) to migrate into the infarcted area to repair and repopulate the region [78]. As such, there has been a research into synthetically inducing epicardial expression of *WT1* and EMT-related genes as it presents an attractive stem cell-based therapy to enable cardiac repair post-myocardial infarction. In the context of miRNA control, Let-7 microRNA expression has been found in the heart and in vitro embryonic stem cell-derived matured cardiomyocytes. Let-7 inhibition post-myocardial infarction has shown to attenuate myocardial remodelling, improve cardiac function, increase the expression of EMT-related genes in the epicardial cells and increase the expression of pluripotency genes *Sox2* and *Oct4* in the epicardial-derived fibroblasts [107, 108]. As such, Let-7 miRNA inhibition could be one of the miRNA-based methods to activate EMT of epicardial stem cells to enable neovascularization and cardiac repair of the infarcted heart.

## Conclusion

The biological importance of miRNAs has been conclusively determined over the past two decades. With roles in stem cell maintenance to the terminal differentiation of cells, these miRNAs behave as molecular rheostats that are highly targetable by synthetic oligonucleotides. In the field of cardiovascular research, significant effort and funding are being poured into regenerative therapy to look for a solution to cardiac regeneration. The reversion of adult cardiomyocytes into a progenitor-like state or activation of the progenitor properties of epicardial WT1+ cells are both potential methods to enable cardiac proliferation. To that end, our increasing understanding of the control miRNA has over pluripotency and proliferation can be utilized. While significant amounts of miRNA modelling have already been carried out in animal models, further validation needs to be carried out in in vitro human cardiomyocyte or 3D cardiac organoid cultures in the near future.

## Data Availability

Not applicable

## Abbreviations

AGO: Argonaute
CHD: Congenital heart disease
miRNA: MicroRNA
MRE: miRNA response element
mRNA: Messenger RNA
pre-miRNA: Precursor miRNA
pri-miRNA: Primary miRNA
RISC: RNA-induced silencing complex
RNAi: RNA interference
TOF: Tetratology of fallot
UTR: Untranslated region
VSD: Ventricular septal defect
